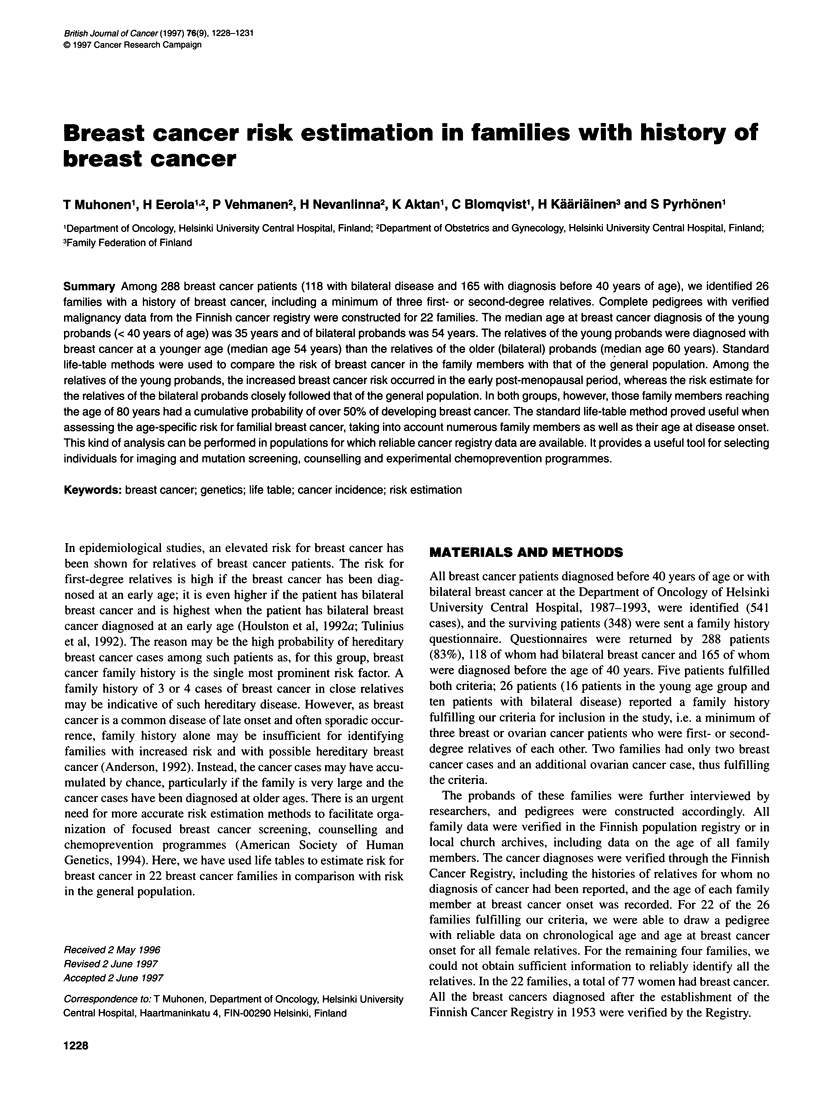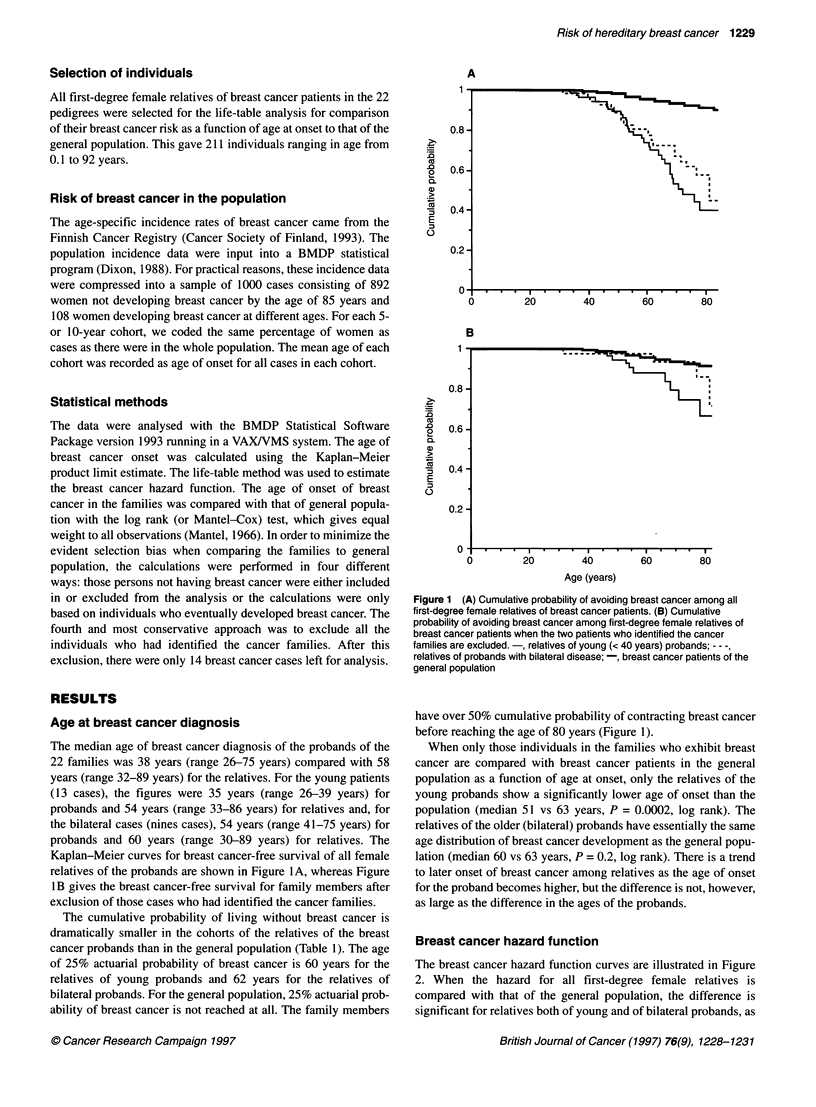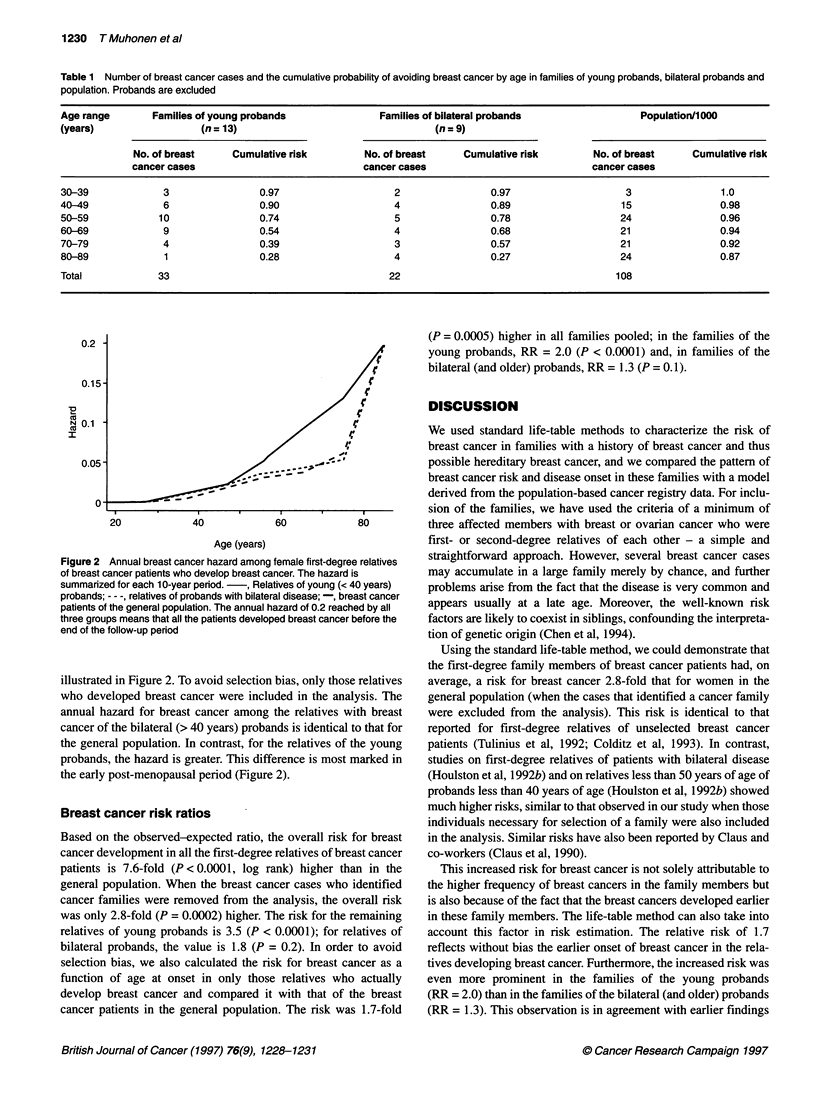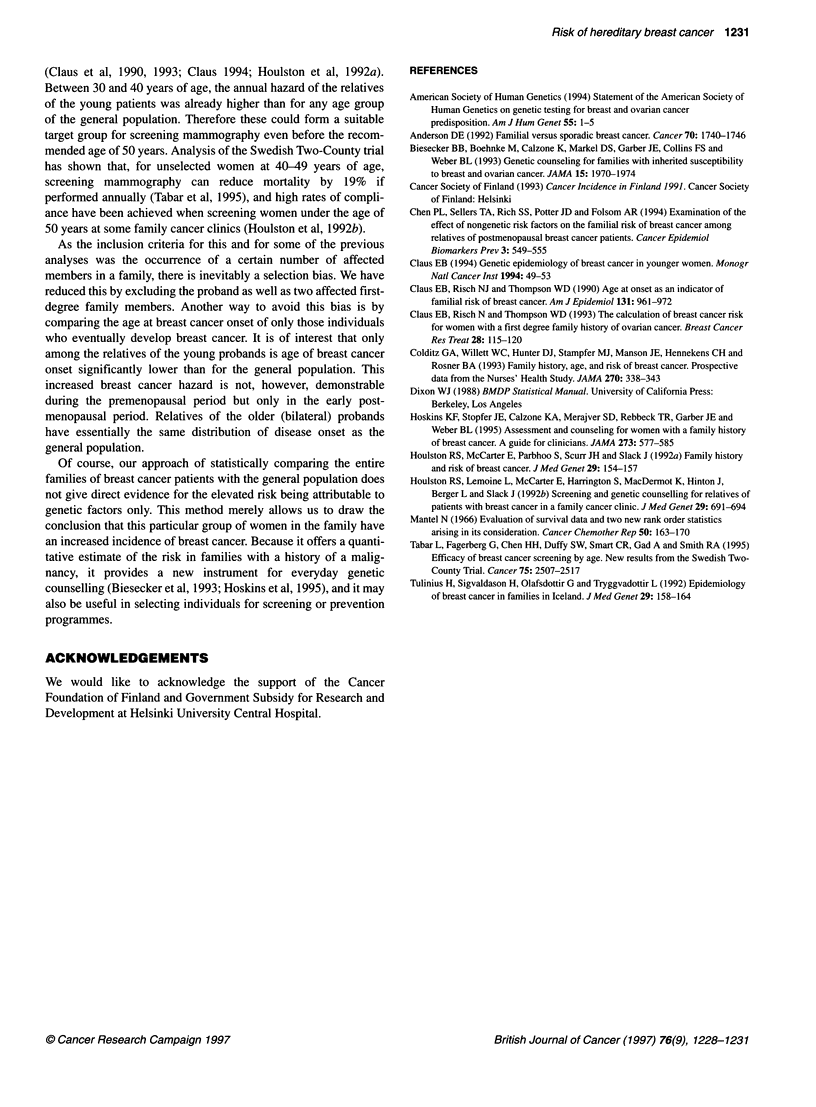# Breast cancer risk estimation in families with history of breast cancer.

**DOI:** 10.1038/bjc.1997.538

**Published:** 1997

**Authors:** T. Muhonen, H. Eerola, P. Vehmanen, H. Nevanlinna, K. Aktan, C. Blomqvist, H. KÃ¤Ã¤riÃ¤inen, S. PyrhÃ¶nen

**Affiliations:** Department of Oncology, Helsinki University Central Hospital, Finland.

## Abstract

Among 288 breast cancer patients (118 with bilateral disease and 165 with diagnosis before 40 years of age), we identified 26 families with a history of breast cancer, including a minimum of three first- or second-degree relatives. Complete pedigrees with verified malignancy data from the Finnish cancer registry were constructed for 22 families. The median age at breast cancer diagnosis of the young probands (< 40 years of age) was 35 years and of bilateral probands was 54 years. The relatives of the young probands were diagnosed with breast cancer at a younger age (median age 54 years) than the relatives of the older (bilateral) probands (median age 60 years). Standard life-table methods were used to compare the risk of breast cancer in the family members with that of the general population. Among the relatives of the young probands, the increased breast cancer risk occurred in the early post-menopausal period, whereas the risk estimate for the relatives of the bilateral probands closely followed that of the general population. In both groups, however, those family members reaching the age of 80 years had a cumulative probability of over 50% of developing breast cancer. The standard life-table method proved useful when assessing the age-specific risk for familial breast cancer, taking into account numerous family members as well as their age at disease onset. This kind of analysis can be performed in populations for which reliable cancer registry data are available. It provides a useful tool for selecting individuals for imaging and mutation screening, counselling and experimental chemoprevention programmes.


British Journal of Cancer (1997) 76(9), 1228–1231
© 1997 Cancer Research Campaign

### Breast cancer risk estimation in families with history of breast cancer

**T Muhonen[1], H Eerola[1,2], P Vehmanen[2], H Nevanlinna[2], K Aktan[1], C Blomqvist[1], H Kääriäinen[3] and S Pyrhönen[1]**

[1]Department of Oncology, Helsinki University Central Hospital, Finland; [2]Department of Obstetrics and Gynecology, Helsinki University Central Hospital, Finland; [3]Family Federation of Finland

**Summary** Among 288 breast cancer patients (118 with bilateral disease and 165 with diagnosis before 40 years of age), we identified 26 families with a history of breast cancer, including a minimum of three first- or second-degree relatives. Complete pedigrees with verified malignancy data from the Finnish cancer registry were constructed for 22 families. The median age at breast cancer diagnosis of the young probands (< 40 years of age) was 35 years and of bilateral probands was 54 years. The relatives of the young probands were diagnosed with breast cancer at a younger age (median age 54 years) than the relatives of the older (bilateral) probands (median age 60 years). Standard life-table methods were used to compare the risk of breast cancer in the family members with that of the general population. Among the relatives of the young probands, the increased breast cancer risk occurred in the early post-menopausal period, whereas the risk estimate for the relatives of the bilateral probands closely followed that of the general population. In both groups, however, those family members reaching the age of 80 years had a cumulative probability of over 50% of developing breast cancer. The standard life-table method proved useful when assessing the age-specific risk for familial breast cancer, taking into account numerous family members as well as their age at disease onset. This kind of analysis can be performed in populations for which reliable cancer registry data are available. It provides a useful tool for selecting individuals for imaging and mutation screening, counselling and experimental chemoprevention programmes.

**Keywords:** breast cancer; genetics; life table; cancer incidence; risk estimation

In epidemiological studies, an elevated risk for breast cancer has been shown for relatives of breast cancer patients. The risk for first-degree relatives is high if the breast cancer has been diagnosed at an early age; it is even higher if the patient has bilateral breast cancer and is highest when the patient has bilateral breast cancer diagnosed at an early age (Houlston et al, 1992*a*; Tulinius et al, 1992). The reason may be the high probability of hereditary breast cancer cases among such patients as, for this group, breast cancer family history is the single most prominent risk factor. A family history of 3 or 4 cases of breast cancer in close relatives may be indicative of such hereditary disease. However, as breast cancer is a common disease of late onset and often sporadic occurrence, family history alone may be insufficient for identifying families with increased risk and with possible hereditary breast cancer (Anderson, 1992). Instead, the cancer cases may have accumulated by chance, particularly if the family is very large and the cancer cases have been diagnosed at older ages. There is an urgent need for more accurate risk estimation methods to facilitate organization of focused breast cancer screening, counselling and chemoprevention programmes (American Society of Human Genetics, 1994). Here, we have used life tables to estimate risk for breast cancer in 22 breast cancer families in comparison with risk in the general population.

*Received 2 May 1996*
*Revised 2 June 1997*
*Accepted 2 June 1997*

*Correspondence to:* T Muhonen, Department of Oncology, Helsinki University Central Hospital, Haartmaninkatu 4, FIN-00290 Helsinki, Finland

### MATERIALS AND METHODS

All breast cancer patients diagnosed before 40 years of age or with bilateral breast cancer at the Department of Oncology of Helsinki University Central Hospital, 1987–1993, were identified (541 cases), and the surviving patients (348) were sent a family history questionnaire. Questionnaires were returned by 288 patients (83%), 118 of whom had bilateral breast cancer and 165 of whom were diagnosed before the age of 40 years. Five patients fulfilled both criteria; 26 patients (16 patients in the young age group and ten patients with bilateral disease) reported a family history fulfilling our criteria for inclusion in the study, i.e. a minimum of three breast or ovarian cancer patients who were first- or second-degree relatives of each other. Two families had only two breast cancer cases and an additional ovarian cancer case, thus fulfilling the criteria.

The probands of these families were further interviewed by researchers, and pedigrees were constructed accordingly. All family data were verified in the Finnish population registry or in local church archives, including data on the age of all family members. The cancer diagnoses were verified through the Finnish Cancer Registry, including the histories of relatives for whom no diagnosis of cancer had been reported, and the age of each family member at breast cancer onset was recorded. For 22 of the 26 families fulfilling our criteria, we were able to draw a pedigree with reliable data on chronological age and age at breast cancer onset for all female relatives. For the remaining four families, we could not obtain sufficient information to reliably identify all the relatives. In the 22 families, a total of 77 women had breast cancer. All the breast cancers diagnosed after the establishment of the Finnish Cancer Registry in 1953 were verified by the Registry.

### Selection of individuals

All first-degree female relatives of breast cancer patients in the 22 pedigrees were selected for the life-table analysis for comparison of their breast cancer risk as a function of age at onset to that of the general population. This gave 211 individuals ranging in age from 0.1 to 92 years.

### Risk of breast cancer in the population

The age-specific incidence rates of breast cancer came from the Finnish Cancer Registry (Cancer Society of Finland, 1993). The population incidence data were input into a BMDP statistical program (Dixon, 1988). For practical reasons, these incidence data were compressed into a sample of 1000 cases consisting of 892 women not developing breast cancer by the age of 85 years and 108 women developing breast cancer at different ages. For each 5- or 10-year cohort, we coded the same percentage of women as cases as there were in the whole population. The mean age of each cohort was recorded as age of onset for all cases in each cohort.

### Statistical methods

The data were analysed with the BMDP Statistical Software Package version 1993 running in a VAX/VMS system. The age of breast cancer onset was calculated using the Kaplan–Meier product limit estimate. The life-table method was used to estimate the breast cancer hazard function. The age of onset of breast cancer in the families was compared with that of general population with the log rank (or Mantel–Cox) test, which gives equal weight to all observations (Mantel, 1966). In order to minimize the evident selection bias when comparing the families to general population, the calculations were performed in four different ways: those persons not having breast cancer were either included in or excluded from the analysis or the calculations were only based on individuals who eventually developed breast cancer. The fourth and most conservative approach was to exclude all the individuals who had identified the cancer families. After this exclusion, there were only 14 breast cancer cases left for analysis.

**Figure 1** (**A**) Cumulative probability of avoiding breast cancer among all first-degree female relatives of breast cancer patients. (**B**) Cumulative probability of avoiding breast cancer among first-degree female relatives of breast cancer patients when the two patients who identified the cancer families are excluded. —, relatives of young (< 40 years) probands; - - -, relatives of probands with bilateral disease; —, breast cancer patients of the general population

### RESULTS

### Age at breast cancer diagnosis

The median age of breast cancer diagnosis of the probands of the 22 families was 38 years (range 26–75 years) compared with 58 years (range 32–89 years) for the relatives. For the young patients (13 cases), the figures were 35 years (range 26–39 years) for probands and 54 years (range 33–86 years) for relatives and, for the bilateral cases (nines cases), 54 years (range 41–75 years) for probands and 60 years (range 30–89 years) for relatives. The Kaplan–Meier curves for breast cancer-free survival of all female relatives of the probands are shown in Figure 1A, whereas Figure 1B gives the breast cancer-free survival for family members after exclusion of those cases who had identified the cancer families.

The cumulative probability of living without breast cancer is dramatically smaller in the cohorts of the relatives of the breast cancer probands than in the general population (Table 1). The age of 25% actuarial probability of breast cancer is 60 years for the relatives of young probands and 62 years for the relatives of bilateral probands. For the general population, 25% actuarial probability of breast cancer is not reached at all. The family members have over 50% cumulative probability of contracting breast cancer before reaching the age of 80 years (Figure 1).

When only those individuals in the families who exhibit breast cancer are compared with breast cancer patients in the general population as a function of age at onset, only the relatives of the young probands show a significantly lower age of onset than the population (median 51 vs 63 years, $P$ = 0.0002, log rank). The relatives of the older (bilateral) probands have essentially the same age distribution of breast cancer development as the general population (median 60 vs 63 years, $P$ = 0.2, log rank). There is a trend to later onset of breast cancer among relatives as the age of onset for the proband becomes higher, but the difference is not, however, as large as the difference in the ages of the probands.

### Breast cancer hazard function

The breast cancer hazard function curves are illustrated in Figure 2. When the hazard for all first-degree female relatives is compared with that of the general population, the difference is significant for relatives both of young and of bilateral probands, as

© Cancer Research Campaign 1997

**Table 1** Number of breast cancer cases and the cumulative probability of avoiding breast cancer by age in families of young probands, bilateral probands and population. Probands are excluded

| Age range (years) | Families of young probands (*n* = 13) | | Families of bilateral probands (*n* = 9) | | Population/1000 | |
|---|---|---|---|---|---|---|
| | No. of breast cancer cases | Cumulative risk | No. of breast cancer cases | Cumulative risk | No. of breast cancer cases | Cumulative risk |
| 30–39 | 3 | 0.97 | 2 | 0.97 | 3 | 1.0 |
| 40–49 | 6 | 0.90 | 4 | 0.89 | 15 | 0.98 |
| 50–59 | 10 | 0.74 | 5 | 0.78 | 24 | 0.96 |
| 60–69 | 9 | 0.54 | 4 | 0.68 | 21 | 0.94 |
| 70–79 | 4 | 0.39 | 3 | 0.57 | 21 | 0.92 |
| 80–89 | 1 | 0.28 | 4 | 0.27 | 24 | 0.87 |
| Total | 33 | | 22 | | 108 | |

**Figure 2** Annual breast cancer hazard among female first-degree relatives of breast cancer patients who develop breast cancer. The hazard is summarized for each 10-year period. ——, Relatives of young (< 40 years) probands; - - -, relatives of probands with bilateral disease; —, breast cancer patients of the general population. The annual hazard of 0.2 reached by all three groups means that all the patients developed breast cancer before the end of the follow-up period illustrated in Figure 2. To avoid selection bias, only those relatives who developed breast cancer were included in the analysis. The annual hazard for breast cancer among the relatives with breast cancer of the bilateral (> 40 years) probands is identical to that for the general population. In contrast, for the relatives of the young probands, the hazard is greater. This difference is most marked in the early post-menopausal period (Figure 2).

### Breast cancer risk ratios

Based on the observed–expected ratio, the overall risk for breast cancer development in all the first-degree relatives of breast cancer patients is 7.6-fold ($P < 0.0001$, log rank) higher than in the general population. When the breast cancer cases who identified cancer families were removed from the analysis, the overall risk was only 2.8-fold ($P = 0.0002$) higher. The risk for the remaining relatives of young probands is 3.5 ($P < 0.0001$); for relatives of bilateral probands, the value is 1.8 ($P = 0.2$). In order to avoid selection bias, we also calculated the risk for breast cancer as a function of age at onset in only those relatives who actually develop breast cancer and compared it with that of the breast cancer patients in the general population. The risk was 1.7-fold ($P = 0.0005$) higher in all families pooled; in the families of the young probands, RR = 2.0 ($P < 0.0001$) and, in families of the bilateral (and older) probands, RR = 1.3 ($P = 0.1$).

### DISCUSSION

We used standard life-table methods to characterize the risk of breast cancer in families with a history of breast cancer and thus possible hereditary breast cancer, and we compared the pattern of breast cancer risk and disease onset in these families with a model derived from the population-based cancer registry data. For inclusion of the families, we have used the criteria of a minimum of three affected members with breast or ovarian cancer who were first- or second-degree relatives of each other – a simple and straightforward approach. However, several breast cancer cases may accumulate in a large family merely by chance, and further problems arise from the fact that the disease is very common and appears usually at a late age. Moreover, the well-known risk factors are likely to coexist in siblings, confounding the interpretation of genetic origin (Chen et al, 1994).

Using the standard life-table method, we could demonstrate that the first-degree family members of breast cancer patients had, on average, a risk for breast cancer 2.8-fold that for women in the general population (when the cases that identified a cancer family were excluded from the analysis). This risk is identical to that reported for first-degree relatives of unselected breast cancer patients (Tulinius et al, 1992; Colditz et al, 1993). In contrast, studies on first-degree relatives of patients with bilateral disease (Houlston et al, 1992*b*) and on relatives less than 50 years of age of probands less than 40 years of age (Houlston et al, 1992*b*) showed much higher risks, similar to that observed in our study when those individuals necessary for selection of a family were also included in the analysis. Similar risks have also been reported by Claus and co-workers (Claus et al, 1990).

This increased risk for breast cancer is not solely attributable to the higher frequency of breast cancers in the family members but is also because of the fact that the breast cancers developed earlier in these family members. The life-table method can also take into account this factor in risk estimation. The relative risk of 1.7 reflects without bias the earlier onset of breast cancer in the relatives developing breast cancer. Furthermore, the increased risk was even more prominent in the families of the young probands (RR = 2.0) than in the families of the bilateral (and older) probands (RR = 1.3). This observation is in agreement with earlier findings

 © Cancer Research Campaign 1997

(Claus et al, 1990, 1993; Claus 1994; Houlston et al, 1992*a*). Between 30 and 40 years of age, the annual hazard of the relatives of the young patients was already higher than for any age group of the general population. Therefore these could form a suitable target group for screening mammography even before the recommended age of 50 years. Analysis of the Swedish Two-County trial has shown that, for unselected women at 40–49 years of age, screening mammography can reduce mortality by 19% if performed annually (Tabar et al, 1995), and high rates of compliance have been achieved when screening women under the age of 50 years at some family cancer clinics (Houlston et al, 1992*b*).

As the inclusion criteria for this and for some of the previous analyses was the occurrence of a certain number of affected members in a family, there is inevitably a selection bias. We have reduced this by excluding the proband as well as two affected first-degree family members. Another way to avoid this bias is by comparing the age at breast cancer onset of only those individuals who eventually develop breast cancer. It is of interest that only among the relatives of the young probands is age of breast cancer onset significantly lower than for the general population. This increased breast cancer hazard is not, however, demonstrable during the premenopausal period but only in the early postmenopausal period. Relatives of the older (bilateral) probands have essentially the same distribution of disease onset as the general population.

Of course, our approach of statistically comparing the entire families of breast cancer patients with the general population does not give direct evidence for the elevated risk being attributable to genetic factors only. This method merely allows us to draw the conclusion that this particular group of women in the family have an increased incidence of breast cancer. Because it offers a quantitative estimate of the risk in families with a history of a malignancy, it provides a new instrument for everyday genetic counselling (Biesecker et al, 1993; Hoskins et al, 1995), and it may also be useful in selecting individuals for screening or prevention programmes.

### ACKNOWLEDGEMENTS

We would like to acknowledge the support of the Cancer Foundation of Finland and Government Subsidy for Research and Development at Helsinki University Central Hospital.

### REFERENCES

American Society of Human Genetics (1994) Statement of the American Society of Human Genetics on genetic testing for breast and ovarian cancer predisposition. *Am J Hum Genet* **55:** 1–5

Anderson DE (1992) Familial versus sporadic breast cancer. *Cancer* **70:** 1740–1746

Biesecker BB, Boehnke M, Calzone K, Markel DS, Garber JE, Collins FS and Weber BL (1993) Genetic counseling for families with inherited susceptibility to breast and ovarian cancer. *JAMA* **15:** 1970–1974

Cancer Society of Finland (1993) *Cancer Incidence in Finland 1991*. Cancer Society of Finland: Helsinki

Chen PL, Sellers TA, Rich SS, Potter JD and Folsom AR (1994) Examination of the effect of nongenetic risk factors on the familial risk of breast cancer among relatives of postmenopausal breast cancer patients. *Cancer Epidemiol Biomarkers Prev* **3:** 549–555

Claus EB (1994) Genetic epidemiology of breast cancer in younger women. *Monogr Natl Cancer Inst* **1994:** 49–53

Claus EB, Risch NJ and Thompson WD (1990) Age at onset as an indicator of familial risk of breast cancer. *Am J Epidemiol* **131:** 961–972

Claus EB, Risch N and Thompson WD (1993) The calculation of breast cancer risk for women with a first degree family history of ovarian cancer. *Breast Cancer Res Treat* **28:** 115–120

Colditz GA, Willett WC, Hunter DJ, Stampfer MJ, Manson JE, Hennekens CH and Rosner BA (1993) Family history, age, and risk of breast cancer. Prospective data from the Nurses' Health Study. *JAMA* **270:** 338–343

Dixon WJ (1988) *BMDP Statistical Manual*. University of California Press: Berkeley, Los Angeles

Hoskins KF, Stopfer JE, Calzone KA, Merajver SD, Rebbeck TR, Garber JE and Weber BL (1995) Assessment and counseling for women with a family history of breast cancer. A guide for clinicians. *JAMA* **273:** 577–585

Houlston RS, McCarter E, Parbhoo S, Scurr JH and Slack J (1992*a*) Family history and risk of breast cancer. *J Med Genet* **29:** 154–157

Houlston RS, Lemoine L, McCarter E, Harrington S, MacDermot K, Hinton J, Berger L and Slack J (1992*b*) Screening and genetic counselling for relatives of patients with breast cancer in a family cancer clinic. *J Med Genet* **29:** 691–694

Mantel N (1966) Evaluation of survival data and two new rank order statistics arising in its consideration. *Cancer Chemother Rep* **50:** 163–170

Tabar L, Fagerberg G, Chen HH, Duffy SW, Smart CR, Gad A and Smith RA (1995) Efficacy of breast cancer screening by age. New results from the Swedish Two-County Trial. *Cancer* **75:** 2507–2517

Tulinius H, Sigvaldason H, Olafsdottir G and Tryggvadottir L (1992) Epidemiology of breast cancer in families in Iceland. *J Med Genet* **29:** 158–164

© Cancer Research Campaign 1997